# Supplementary material for: Learning receptive field properties of complex cells in V1
Source: PLoS Comput Biol. 2021 Mar 2;17(3):e1007957. doi: 10.1371/journal.pcbi.1007957 (PMC7954310; doi:10.1371/journal.pcbi.1007957)
Supplement: S1 Appendix — This document gives details on learning complex cells using jittered natural images instead of a natural video. (PDF) [file pcbi.1007957.s001.pdf]

S1 Appendix: complex cells can also be learned using jittered images

Using Natural images with jitter

50 randomly selected 1024×1536 pixel images of calibrated natural scenes from van Hateren's dataset [1] were used to train the simple cells in the model. However, jittered image patches from these 50 natural scenes also provide enough temporal information such that complex cells can be learned.

For natural visual stimuli with temporal information, such as videos, the changing content between subsequent frames in one fixed region over a very short time period is very similar except for some translations or shifts in position, as results from the movement of an object or of the eyes themselves. Therefore, the temporal information in a natural video is similar to sequences of translated images, as used to investigate temporal slowness learning [2]. Here, we simply use a sequence of natural images with a random spatial jitter to incorporate temporal information.

To represent the image patches of temporal stimuli in one location over a short time period, we take N image patches randomly jittered around that location. More specifically, for a 1024×1536 pixel natural image from the natural image data set, a random pixel location, (i_x, i_y), is chosen and then N image patches of size $M \times M$ whose centers are within a $0.4M$ pixel distance of (i_x, i_y) are randomly selected. For the N chosen image patches, the bottom two-layer model generates N sets of simple cell responses. Since the N image patches contain similar features with spatial shifts, the neural activities of simple cells in response to the N image patches will also contain spatial phase information. Using the concept of the trace rule, where the response is determined by the current and past responses [3], the average of N sets of simple cell responses is then used as the input to the complex cells; i.e., $\mathbf{x}^{\mathrm{C}} = \langle \mathbf{r}^{\mathrm{S}} \rangle$.

The same simple cells trained on the natural image dataset (van Hateren's dataset [1]) are used here. The connection between simple and complex cells is learned using the same learning rule in the paper. Same parameter values are used for the modified NBCM rule where possible. There are 4×10^6 epochs in the training process. The learning rates for weights and threshold are $\eta_a = 10^{-3}$ and $\eta_\theta = 10^{-3}$, respectively. The weight regularization constant, $\gamma_a = 10^{-4}$ and the maximal connection weight, $a_{2,\max} = 1$. For the parameters in the divisive normalization, α is 0.01 and $\beta = 12$. The number of jittered images in each iteration, N, is taken to be 3, 5, and 8 to investigate the effects of N.

Next, we show that the model can also learn complex cells properties that matches experimental data using jittered natural images instead of natural video.

Number of spatially jittered image patches determines the level of spatial phase invariance of model complex cells

Temporal information is prevalent in the real world, so it is important to consider its effect on the learning of complex cell receptive fields. Since the image patches around a location tend to have similar features but differ slightly in orientation, position, and size, the average responses of simple cells for these adjacent image patches integrate invariance and pass it to complex cells. Spatial phase invariance arises in the model complex cell due to presentation of spatially jittered versions of the same image during a learning batch. We investigated how the degree of spatial phase invariance depends on the number of jittered image patches ($N = 3$, 5, and 8) for the modified NBCM rule model.

Intuitively, the larger N is, the more spatially phase invariant the model complex cells will be because more image patches will lead to a greater sampling of different spatial phases of the same features. This is consistent with the simulation results, as can be seen in Fig-A 1, which shows that more complex cells have small F_1/F_0 ratio when N increases, indicating greater spatial phase invariance.

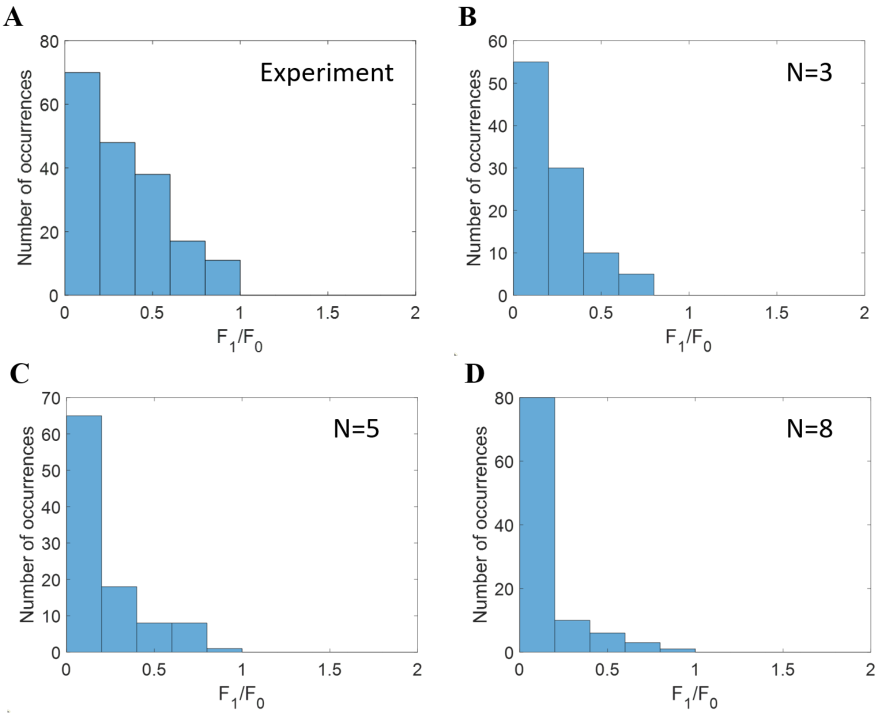

Fig-A 1: **Histograms of F_1/F_0 for models based on the modified NBCM rule.** (A) Experimental complex cells [4]. Model complex cells learned with (B) $N = 3$, (C) $N = 5$, and (D) $N = 8$.

Compared with the data for complex cells in an experimental study [4] shown in Fig-A 1A, the histogram of F_1/F_0 for $N = 5$ (Fig-A 1B) is the most similar because it has a similar diversity for cells with $F_1/F_0 < 1$ compared with $N = 3$ and $N = 8$.

We also investigate the diversity of how tightly or broadly cells are tuned to orientation in the population of model complex cells. This is assessed by measuring orientation tuning bandwidth of response to drifting gratings. Fig-A 2 shows the histogram of half-bandwidth for experimental and model data (only data points with $F_1/F_0 <= 1$ are included). The histograms in Fig-A 2 show that the distribution of orientation bandwidth has a modal value similar to experimental findings [4], but models with different values of N change the proportion of cells with small orientation bandwidth. As N increases, more complex cells have small orientation bandwidth (i.e., stronger orientation selective).

Combining both Fig-A 1 and 2, as N increases, model cells become more invariant to spatial phases and more selective to orientations at the same time. One possible reason is because more features with similar orientations but different phases tend to occur in one iteration as more random jittered patches are used, which makes the model to pool simple cells with similar orientations but different phases. Therefore, more model cells have small F_1/F_0 ratio and orientation bandwidth as N increases.

Population statistics compared with experimental data

To quantitatively compare our results for the learned model with these experimental results, population statistics are analyzed using the three measures of tuning used in the experimental study orientation breadth, spatial frequency breadth, and spatial phase breadth. Given the better match to experimental data (Fig-A 1 and Fig-A 2), the data set for $N = 5$ is used here. Learned complex cells are diverse, similar to examples shown in Fig 8 of the paper. Comparisons between model and experimental data [5] are shown in Fig-A 3.

Fig-A 3A shows that most cells for both model and experimental data are highly tuned to orientation (orientation breadth $< 45°$), but experimental complex cells have a somewhat broader distribution of orientation breaths than the model complex cells. The overlap between the histograms of model and experimental data accounts for 85.2% of the histograms spanned by both model and experimental data.

Fig-A 3B shows that model data has similar range of tuning to spatial frequency as experimental data. The spatial frequency breadth of the model displayed in the figure is scaled to have the same maximum

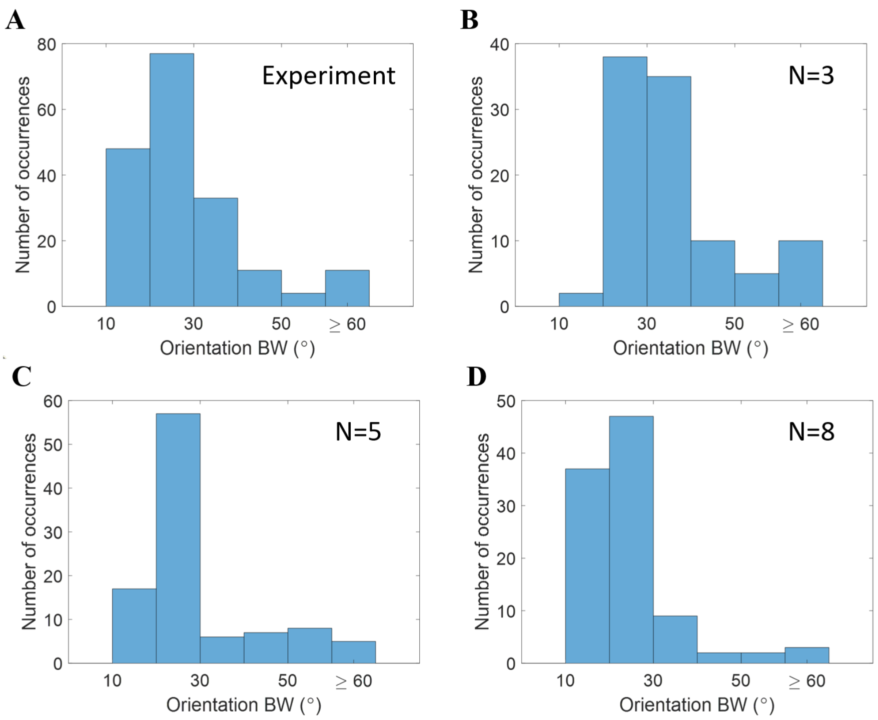

Fig-A 2: **Histograms of half-bandwidth for models based on the modified NBCM rule.** (A) Experimental complex cells [4]. (B) $N = 3$. (C) $N = 5$. (D) $N = 8$.

as the experimental data. The overlap between the histograms of model and experimental data accounts for 96.7% of the histograms spanned by both model and experimental data.

Fig-A 3C shows that both model and experimental data cover a wide range of spatial phase tuning, except that the model data has more complex cells with spatial phase breadth around 270 degrees. The overlap between the histograms of model and experimental data accounts for 68.5% of the histograms spanned by both model and experimental data.

Overall, using jittered natural images, the model can still account for the diversity of complex cells found in the experimental study of Almasi et al. [5]. Despite some discrepancies in the histograms of these three measures of tuning, the model can capture the trends of the distributions.

References

[1] Van Hateren JH, Van Der Schaaf A. Independent component filters of natural images compared with simple cells in primary visual cortex. Proc Biol Sci. 1998;265(1394):359–366.

[2] Berkes P, Wiskott L. Slow feature analysis yields a rich repertoire of complex cell properties. J Vision. 2005;5(6):579–602.

[3] Földiák P. Learning invariance from transformation sequences. Neural Comput. 1991;3(2):194–200.

[4] Ringach DL, Shapley RM, Hawken MJ. Orientation selectivity in macaque V1: diversity and laminar dependence. J Neurosci. 2002;22(13):5639–5651.

[5] Almasi A, Meffin H, Cloherty SL, Wong Y, Yunzab M, Ibbotson MR. Mechanisms of Feature Selectivity and Invariance in Primary Visual Cortex. Cereb. Cortex. 2020;30(9):5067–5087.

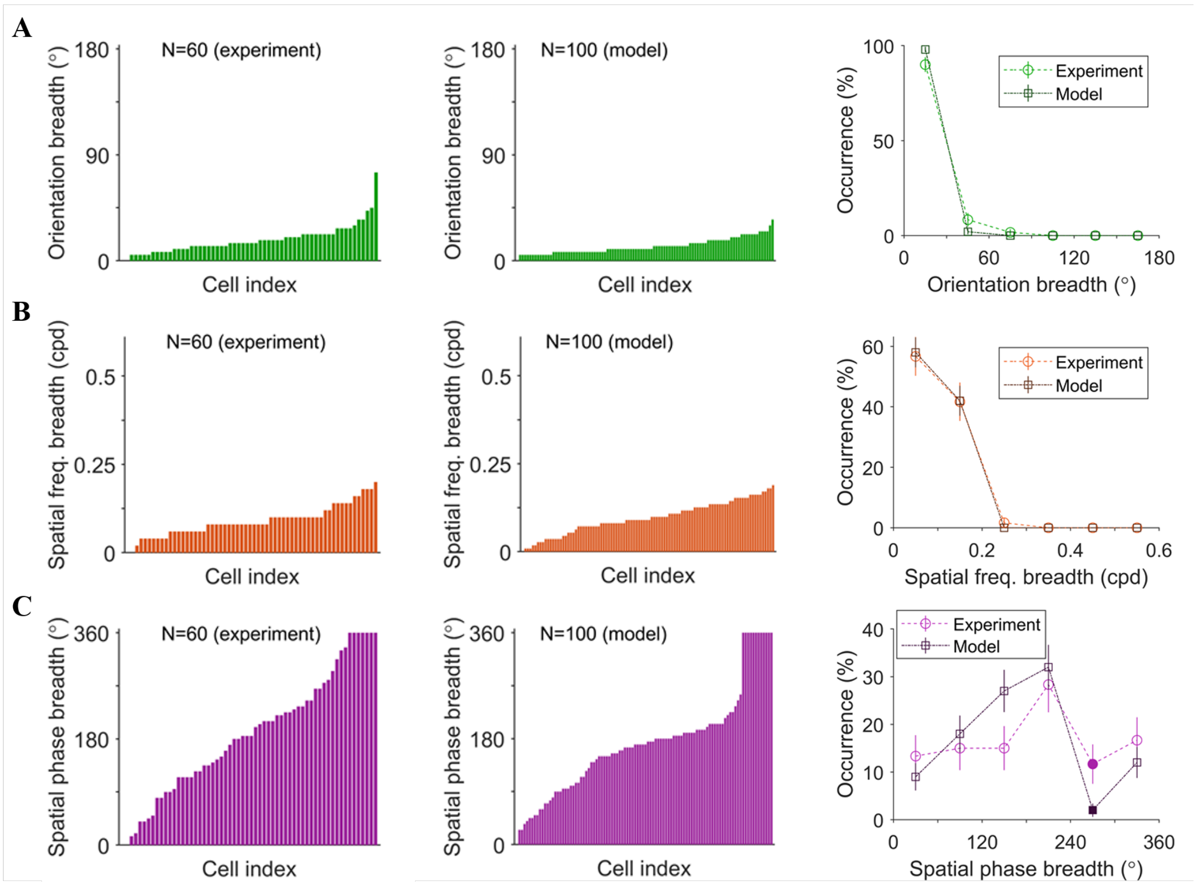

Fig-A 3: **Comparison between experimental data [5] (left) and model data trained using the modified NBCM rule (middle)**. Right: histograms, where filled points indicate differences that are significant (p-value< 0.05; Welch's t-test). (A) Orientation breadth (°). (B) Spatial frequency breadth (circles per degree). (C) Spatial phase breadth (°).
