# Supplementary material for: Learning receptive field properties of complex cells in V1
Source: PLoS Comput Biol. 2021 Mar 2;17(3):e1007957. doi: 10.1371/journal.pcbi.1007957 (PMC7954310; doi:10.1371/journal.pcbi.1007957)
Supplement: S2 Appendix — This document gives details on investigating efficient coding for learning complex cells. (PDF) [file pcbi.1007957.s002.pdf]

S2 Appendix: Can complex cells be learned by efficient coding?

Methods

Model dynamics

For investigating whether efficient coding principle can learn complex cells, the same efficient coding model of LGN-V1 pathway is applied to simple-complex cells connection. A four-layer model was built to describe the activities of lateral geniculate nucleus (LGN) cells (first layer), V1 simple cells (second layer), intermediate cells (third layer), and V1 complex cells (fourth layer). The intermediate cells take simple cell responses and provide input for complex cells, which separates the computations of simple and complex cells, and helps us investigate efficient coding for complex cells much easier.

The dynamics of LGN and simple cells are the same as described in the paper. Similarly, the top two layers implement a second efficient coding model, but complex cells receiving inputs from simple cells. The dynamics of the top two layers are given by

$$\begin{aligned} \tau_{\mathrm{I}} \dot{\mathbf{v}}^{\mathrm{I}} &= -\mathbf{v}^{\mathrm{I}} + \mathbf{x}^{\mathrm{I}} + \mathbf{A}_{\mathrm{C}}^{\mathrm{I}} \mathbf{r}^{\mathrm{C}} + r_{\mathrm{b,I}} \\ \mathbf{r}^{\mathrm{I}} &= \max(\mathbf{v}^{\mathrm{I}}, 0), \end{aligned} \tag{1}$$

and

$$\begin{aligned} \tau_{\mathrm{C}} \dot{\mathbf{v}}^{\mathrm{C}} &= -\left(\mathbf{v}^{\mathrm{C}} - \mathbf{v}_{\mathrm{leak}}^{\mathrm{C}}\right) + \mathbf{A}_{\mathrm{I}}^{\mathrm{C}\,T} \mathbf{r}^{\mathrm{I}} + \mathbf{r}^{\mathrm{C}} \\ \mathbf{r}^{\mathrm{C}} &= \max(\mathbf{v}^{\mathrm{C}} - \lambda_{\mathrm{C}}, 0), \end{aligned} \tag{2}$$

where τ_{I}, $\mathbf{x}^{\mathrm{I}}$, $\mathbf{v}^{\mathrm{I}}$, $\mathbf{r}^{\mathrm{I}}$ and $r_{\mathrm{b,I}}$ are the time constant, input, membrane potential, firing rate, and background firing rate of intermediate cells in the third layer, τ_{C}, $\mathbf{v}^{\mathrm{C}}$, $\mathbf{r}^{\mathrm{C}}$ and λ_{C} are the time constant, membrane potentials, firing rates and firing threshold for complex cells in the fourth layer. $\mathbf{v}_{\mathrm{leak}}^{\mathrm{C}}$ represents the change of membrane potential for complex cells caused by leakage currents. $\mathbf{A}_{\mathrm{I}}^{\mathrm{C}}$ represents the feedforward connection from intermediate cells to complex cells. Weights $\mathbf{A}_{\mathrm{I}}^{\mathrm{C}}$ are taken to be excitatory here so there are no inhibitory connections from intermediate cells to complex cells. The inputs to intermediate cells are the responses of simple cells so the excitatory connections between intermediate cells and complex cells indicate which simple cells are pooled for complex cells and how they are weighted. Introducing intermediate cells separates the process of computing simple and complex cell responses so that we can simply investigate efficient coding for complex cells. $\mathbf{A}_{\mathrm{C}}^{\mathrm{I}}$ represents the feedback connection from complex cells to simple cells; i.e., $\mathbf{A}_{\mathrm{C}}^{\mathrm{I}} = \mathbf{A}_{\mathrm{C}}^{\mathrm{I},+} + \mathbf{A}_{\mathrm{C}}^{\mathrm{I},-}$, where $\mathbf{A}_{\mathrm{C}}^{\mathrm{I},+}$ are the excitatory connections from complex cells to simple cells and $\mathbf{A}_{\mathrm{C}}^{\mathrm{I},-}$ represent the inhibitory connections.

Learning simple-complex cell connections

Because the bottom two layers remain unchanged, the learning rule of LGN-simple cells connections stay the same.

The top two layers implement a similar model to the bottom two layers. The connections between simple and complex cells are implemented via intermediate cells. The learning rule comes from efficient coding and is similar to LGN-simple cells connections, as given by

$$\begin{aligned} \Delta \mathbf{A}_{\mathrm{I}}^{\mathrm{C}} &= \eta_2 \left(\langle (\mathbf{r}^{\mathrm{L}} - r_{\mathrm{b,I}}) \mathbf{r}^{\mathrm{I}\,T} \rangle - \gamma_2 \mathbf{A}_{\mathrm{I}}^{\mathrm{C}} \right) \\ \Delta \mathbf{A}_{\mathrm{C}}^{\mathrm{I},+} &= -\eta_2 \left(\langle (\mathbf{r}^{\mathrm{L}} - r_{\mathrm{b,I}}) \mathbf{r}^{\mathrm{I}\,T} \rangle - \gamma_2 \mathbf{A}_{\mathrm{C}}^{\mathrm{I},+} \right) \\ \Delta \mathbf{A}_{\mathrm{C}}^{\mathrm{I},-} &= -\eta_2 \left(\langle (\mathbf{r}^{\mathrm{L}} - r_{\mathrm{b,I}}) \mathbf{r}^{\mathrm{I}\,T} \rangle - \gamma_2 \mathbf{A}_{\mathrm{C}}^{\mathrm{I},-} \right), \end{aligned} \tag{3}$$

where η_2 and γ_2 are the learning rate and weight regulation constant, respectively. In addition, the maximal synaptic weight allowed is $a_{2,\max}$.

Input and training

The natural images are used as the input to the first layer and simple cell responses generated in the second layer are the input to the top two layers where a rule based on efficient coding is used to learn the subspace of complex cells. The input to intermediate cells is the average of simple cell responses over natural image with jitter; i.e., $\mathbf{x}^{\mathrm{I}} = \langle \mathbf{r}^{\mathrm{S}} \rangle$.

During the training process, the number of image patches for jitter, N, is set to 20 and there are 100 simple cells, 100 intermediate cells and 100 complex cells. LGN-simple cell connections are taken from the learned values in the paper. The top two layers are then trained to learn complex cells. Similarly, in accord with results in [1], the feedforward excitatory (or inhibitory) connections converge to the opposite of the feedback inhibitory (or excitatory) connection, so it can be reasonably assumed that $\mathbf{A}_{\mathrm{C}}^{\mathrm{I},-} = -\mathbf{A}_{\mathrm{I}}^{\mathrm{C}}$ and $\mathbf{A}_{\mathrm{C}}^{\mathrm{I},+} = \mathbf{0}$ given that $\mathbf{A}_{\mathrm{I}}^{\mathrm{C}}$ is taken to be excitatory here. The maximal weight allowed for connections between simple and complex cells (via intermediate cells), $a_{2,\max}$, is 0.3. The learning rate, η_2, and the weight regulation constant, γ_2, are set to 0.5 and 0.0001, respectively. The sparsity level of complex cells, λ_{C}, is set to 0. The learning process for complex cells runs for $100,000$ epochs.

The linearised efficient coding model

After learning, the values of elements in the weight matrix $\mathbf{A}_{\mathrm{C}}^{\mathrm{I}}$ indicate how simple cells are pooled by complex cells. In order to investigate the subspace pooled by each model complex cell, a control model of complex cells that linearly sums over the responses of pooled simple cells using learned connections is used, as given by

$$\mathbf{r}^{\mathrm{C}} = \mathbf{A}_{\mathrm{I}}^{\mathrm{C}^T} \mathbf{r}^{\mathrm{S}}. \tag{4}$$

The control model will be called the linearised efficient coding model throughout this appendix.

The linearised efficient coding model uses the connection weights learned by our efficient coding model, but it uses a different method, linear summation, of computing complex cell responses compared with the efficient coding model. Two important questions that arise here are (1) does the subspace contain simple cells that are similar to those observed experimentally and that have similar orientations but different spatial phases and (2) does the principle of efficient coding reduce spatial phase invariance for complex cells?

Results: efficient coding for complex cells trained on natural images with jitter fails to explain complex cell properties

Simulation results show that efficient coding model of complex cells can pool simple cells with similar orientations but a wide range of spatial phase preferences, but the network dynamics of efficient coding suppresses model cell responses so that spatial phase invariance cannot be generated. After training the model using natural images with jitter, most connection weights reduce to small values while only a few weights are significant, i.e., with values larger than 0.12 (the upper bound, $a_{2,\max}$, is 0.3).

Examples of model complex cells

In this section, we show examples of model complex cells trained on simple cell responses using natural images with temporal information. We demonstrate that even though efficient coding can pool simple cells with different spatial phase preferences that are sufficient to contribute to the spatial phase invariance, the model complex cells display no spatial phase invariance. The following examples account for the diversity observed in the population of model complex cells.

Fig-A 1A shows an example of complex cell, C22, where the simple cells in the subspace have two distinct orientations. As seen from Fig-A 1B, different simple cells are tuned to different spatial phases when sinusoidal gratings with preferred orientation and frequency are used as the input stimuli to the model. The subspace has two orientations, with the first (S64), third (S69), and fifth (S20) simple cells being more dominant as seen from their much stronger spatial phase tuning curves than the second (S75) and fourth (S99) simple cells. Though simple cells in the subspace do not cover all 360°) region of spatial phase, they contribute to a large extent of spatial phase invariance as can be seen from the spatial phase tuning curve of the linearised efficient coding model that has $F_1/F_0 = 0.734$. However, the model complex cell exhibits very limited spatial phase invariance with $F_1/F_0 = 1.38$.

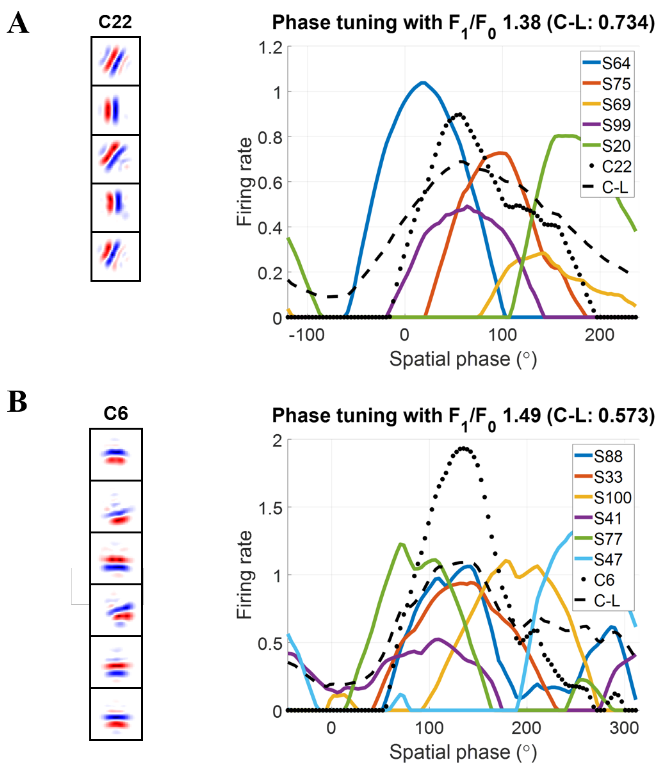

Fig-A 1: **Examples of model complex cells based on efficient coding model.** Left: each block is a 16×16 synaptic field for simple cells in the subspace and values in each block are normalized to the range $[-1\ 1]$ when plotting the figure. Right: spatial phase tuning curves. Solid lines are for simple cells in the subspace. Dotted line is for complex cell. S represents simple cell and the following number is the index of the simple cell. (A) Complex cell C22. (B) Complex cell C6.

Fig-A 1B shows another example of complex cell, C6, that lies between the first and second examples: the simple cells in the subspace have different but similar orientations. As seen from Fig-A 1B, for the sinusoidal gratings with preferred orientation and frequency and different spatial phases, these simple cells in the subspace have different spatial phase tuning curves with different spatial phase preferences, which cover almost the whole 360° of spatial phase. Therefore, the F_1/F_0 ratio is 0.573 for the linearised efficient coding model, which indicates that the subspace is sufficient to generate spatial phase invariance. However, the spatial phase tuning curve of the model complex cell is limited to a subset of 360° with $F_1/F_0 = 1.49$.

Histogram of F_1/F_0 implies that efficient coding makes model complex cells 'simple'

The histogram of F_1/F_0 for the linearised efficient coding model (Fig-A 2C) is closer to experimental data (Fig-A 2A) and shows a distribution centered at around 0.6 with most values smaller than 1, suggesting that the learned subspace of the model complex cell is actually sufficient to generate spatial phase invariance if the model is just a weighted linear summation of simple cell responses. However, the distribution for the model complex cells (Fig-A 2B) is skewed toward 2, indicating that most model complex cells are actually categorized as simple cells according to their values of F_1/F_0. Therefore, efficient coding makes model complex cells 'simple' by the principle of efficient coding itself because of suppressing model cell activities.

The above indicates that efficient coding on simple cell responses using natural images with temporal information can pool simple cells to form the subspace of complex cells. However, the competition between complex cells brought about by efficient coding suppresses complex cell responses such that they do not show spatial phase invariance.

Different model complex cells may pool the same simple cell, but complex cells might lose the spatial phase invariance brought by the simple cell when complex cells are competing with each other to represent

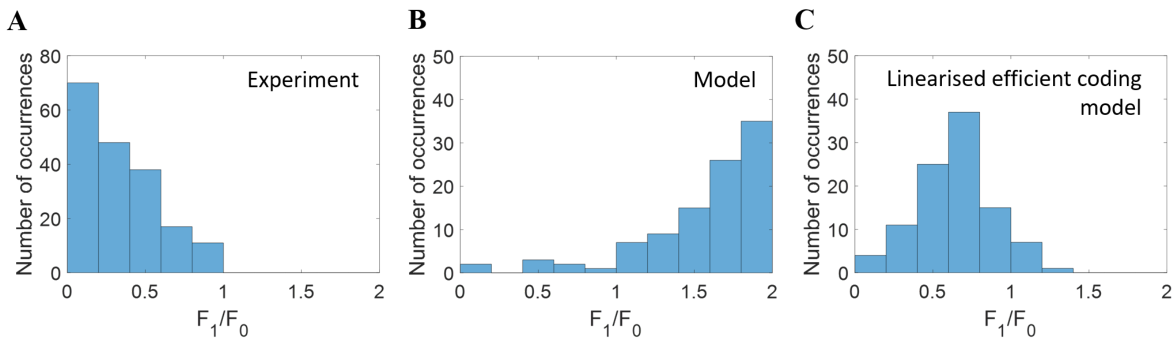

Fig-A 2: **Histograms of F_1/F_0 of the model based on efficient coding.** (A) Experimental complex cells [2]. (B) Model complex cells. (C) Linearised efficient coding model of complex cells.

the simple cell response. Therefore, the spatial phase tuning curves of such model complex cells are much narrower than the linearised efficient coding model, which implies that efficient coding help model complex cells selectively pool simple cells, but that the competition introduced by efficient coding suppress model cell responses such that they behave like simple cells.

References

[1] Lian Y, Meffin H, Grayden DB, Kameneva T, Burkitt AN. Towards a biologically plausible model of LGN-V1 pathways based on efficient coding. Front Neural Circuits. 2019;13:13.

[2] Ringach DL, Shapley RM, Hawken MJ. Orientation selectivity in macaque V1: diversity and laminar dependence. J Neurosci. 2002;22(13):5639–5651.
