# Supplementary figures and images for: Learning receptive field properties of complex cells in V1
Source: PLoS Comput Biol. 2021 Mar 2;17(3):e1007957. doi: 10.1371/journal.pcbi.1007957 (PMC7954310; doi:10.1371/journal.pcbi.1007957)

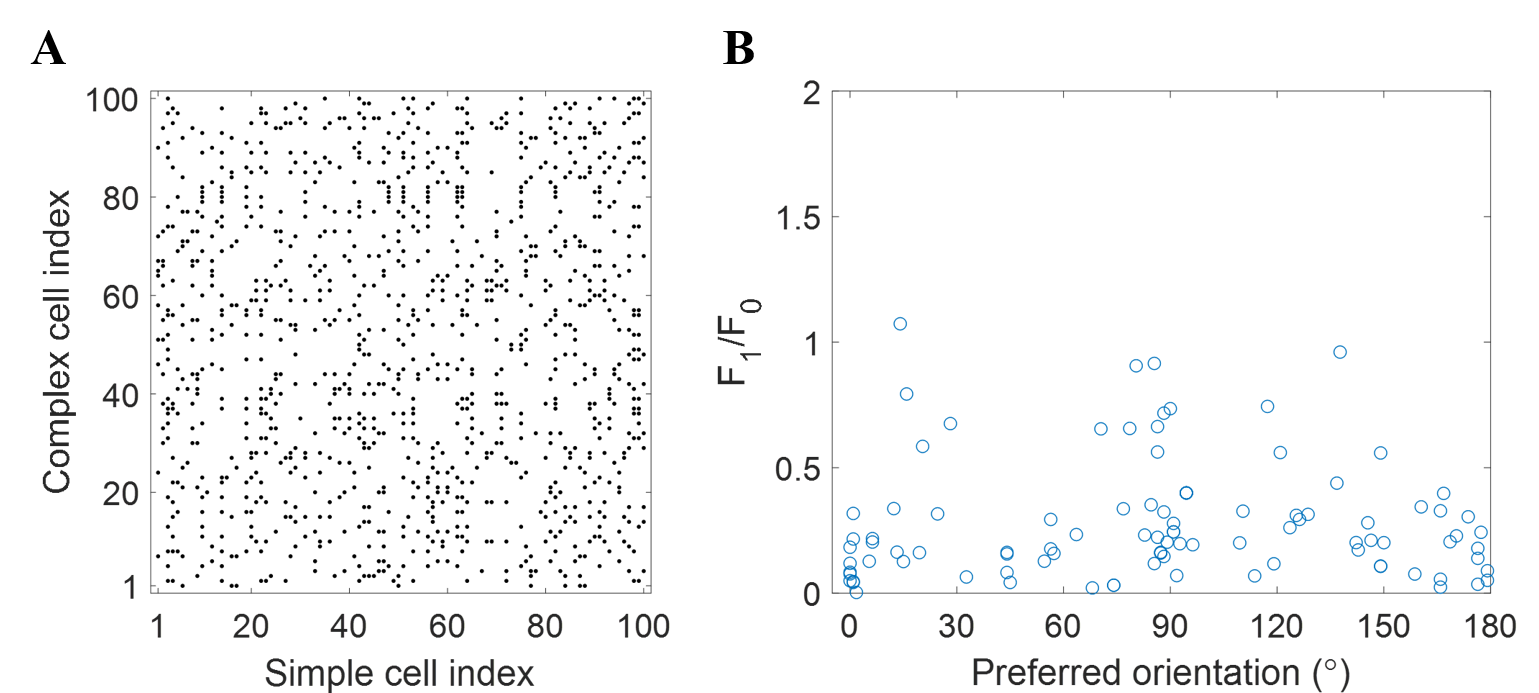

Supplement: S1 Fig — (TIF) [file pcbi.1007957.s003.tif]
