# Supplementary material for: Learning receptive field properties of complex cells in V1
Source: PLoS Comput Biol. 2021 Mar 2;17(3):e1007957. doi: 10.1371/journal.pcbi.1007957 (PMC7954310; doi:10.1371/journal.pcbi.1007957)
Supplement: Attachment — Submitted filename: Response Letter.docx [file pcbi.1007957.s004.docx]

Department of Biomedical Engineering

The University of Melbourne

Carlton, VIC 3053, Australia

[yanbo.lian@unimelb.edu.au](mailto:yanbo.lian@unimelb.edu.au)

22nd Sep. 2020

**Re: Response letter – PLOS Computational Biology (PCOMPBIOL-D-20-00804)**

Dear Deputy Editor and Reviewers,

Thank you for your comments on our manuscript, “Learning receptive field properties of complex cells in V1”, which are very constructive and have helped to improve the paper and provide greater impact of our work to the community.

In response to reviewer comments, we substantially revised the manuscript to present results on the model trained with natural videos instead of jittered natural images (which are now reported in S1 Appendix).

**In response to the comments of Reviewer #1**:

1) Clarify the quantitative comparison of the results from the model with the biological data:

- When comparing the model data and experimental data by Almasi et al. 2020, the Welch’s t-test was performed when generating the histograms in Figure 9. Corresponding changes in the manuscript:
  - Line 465-467: *‘Histograms of these three measures for both model and experimental data, along with the statistical comparison using Welch's t-test, are generated to investigate the performance of the model.’*
  - The histograms on the right of Figure 9 include results of statistical tests.
  - Caption of Figure 9: *‘Right: histograms, where filled points indicate differences that are significant (p-value<0.05; Welch’s t-test).’*
- We also used the percentage, *[overlap between histograms of model and experimental data] / [histogram spanned by both model and experimental]*, to quantitatively show how well the model data represents experimental data. Corresponding changes in the manuscript:
  - Line 605-607: *‘In general, the overlap between the histograms of model and experimental data accounts for 88.7% of the histograms spanned by both model and experimental data.’*
  - Line 613-615: *‘In general, the overlap between the histograms of model and experimental data accounts for 93.6% of the histograms spanned by both model and experimental data.’*
  - Line 618-620: *‘In general, the overlap between the histograms of model and experimental data accounts for 60.4% of the histograms spanned by both model and experimental data.’*

2) Identify the key aspect of the model

- We have explained that the normalization gain, $\beta$, will change the diversity of learned complex cells. This result is shown explicitly in Figure 5, which illustrates the transition from BCM to NBCM. Corresponding changes in the manuscript:
  - Line 523-536: *‘In comparison with model complex cells learned by the modified BCM rule, the modified NBCM rule forces the model to learn different complex cells with different orientations such that there is little overlap or repetition in pronounced simple cell inputs across the complex cell population. The scatter plots of simple-complex cell connections with different values of normalization gain, β, are given in the left column of Fig 5B, C, and D, which shows that simple-complex connections are considerably more diverse than those given by the modified BCM learning rule (Fig 5A). Dots in the figure appear more randomly assigned to complex cells, and there is much less evidence of vertical lines, such as those that appear in Fig 5A. Furthermore, the scatter plot of F1/F0 vs. preferred orientation (right column of Fig 5B, C and D) covers a much wider range compared with Fig 5A, including all orientations (0 to 180^◦^) and range of spatial phase tuning from complete invariance to moderate tuning (F1/F0 from 0 to 1). This indicates that the tuning properties are more diverse than those obtained with the modified BCM rule.’*
- We also added a subsection ‘*Normalization gain determines the level of competition*’ to identify the key features of the modified NBCM learning rule and the transition from repetitious complex cells to diverse complex cells. Corresponding changes in the manuscript:
  - Line 539-569: *‘Fig 5 shows that the modified NBCM rule …’*

3) From the analogy with the NIM and results by Almasi et al. 2020, how to justify the model results (notably the non-linearities)

- The NIM method used by Almasi et al. 2020 recovers the spatial filters with corresponding non-linearities that account for the responses of complex cells. Simple cells can be approximately characterized by a spatial filter with a one-sided non-linearity. In our model of complex cells, the learning rule selects simple cells with different phases, spatial locations, orientations, and spatial frequencies into the subspace of a complex cell such that the combined effect of pooled simple cells can be approximately characterized by spatial filters with more complex non-linearities (the level of non-linearities also depends on the pooled simple cells). Therefore, after using NIM, diverse non-linearities of complex cells can be obtained.

4) Simplify the presentation of the model by summarizing the different heuristics, and to highlight the most important factors which lead to the emergence of complex cells properties in your neuronal model

- See response to 2) above.

5) Make sure the code will be made available at publication time

- The code implementing the model has been made available in the repository given in the manuscript. Corresponding changes in the manuscript:
  - Line 155-156: *‘The code is available at https://github.com/lianyunke/Learning-Receptive-Field-Properties-of-Complex-Cells-in-V1.’*

6) The learning rate, $\eta_{1}$, is 3 - this value seems very high compared to other studies

- This value of learning rate does not cause unstable issues for the learning of simple cells. One reason might be because batch learning with 100 image patches is used in each iteration when training simple cells.

7) Other minor points

- The typos and syntax errors noted by the reviewer have been corrected.

**In response to the comments of Reviewer #2**:

8) Comparison with experimental histograms is currently very qualitative. This comparison needs to be done with statistical tests

- See response to 1) above.

9) The orientation tuning width for complex cells is measured as a variance in the preferred orientation of subunits. However, there is also finite orientation tuning for each subunit. This orientation tuning width can be extracted by taking a Fourier transform of the spatial profile of each subunit.

- In our previous version of the manuscript, we did not make it clear that we used sine gratings to obtain the orientation tuning curve and then compute the preferred orientation and orientation bandwidth (as in Ringach et al. 2002). In the revised manuscript, it is made explicit that we use sine gratings to measure the orientation tuning properties. Corresponding changes in the manuscript:
  - Line 380-382: *‘Similar to measuring spatial phase tuning, an exhaustive search for each model complex cell is first conducted to find the preferred spatial frequency, orientation, and spatial phase of the sinusoidal gratings that generates maximal complex cell response.’*
  - Line 388-390: *‘This orientation tuning curve is then used to compute the preferred orientation and orientation bandwidth as given below.’*

10) It would be better to use natural videos rather than jittered natural images

- We used a natural video (‘Walking in the Woods’, <https://youtu.be/K-Vr2bSMU7o>) with a resolution of 1080x1920 pixels and frame rate 24 to learn complex cells. We added a new section ‘*Natural video*’. Corresponding changes in the manuscript:
  - Line 161-184: *‘For natural visual stimuli with temporal information …’*
- Using natural video resulted in updating all figures in the Results section.
- The results using jittered natural images of van Hateren’s dataset from the previous version of the manuscript are put into S1 Appendix.

11) There are too many figures in the manuscript

- Removed Figure 3 and 5 in the previous manuscript.
- Generated updated figures for the Results section, since natural videos were used for training as suggested by the Reviewer #2.
- Examples of complex cells are combined into one figure.
- The revised manuscript contains 9 figures.

12) Other minor points

- The typos on lines 208 and 196 (in the previous manuscript) noted by the reviewer have been corrected.

To incorporate the changes suggested by the reviewers, the manuscript has been significantly modified. The following list details the changes made in the manuscript:

- Figures
  - Removed Figures 3 and 5 in the previous manuscript.
  - Generated updated figures for the Results section using natural videos for training, as suggested by Reviewer #2.
- Methods
  - Modified the subsection ‘*Measuring orientation tuning*’ to make it clear how orientation tuning curve is obtained.
  - Added a subsection ‘*Natural video*’ under section ‘Input’ to discuss the video data used for learning complex cells.
  - Minor changes of model parameters of complex cells to improve the model convergence. The difference in results from the previous manuscript are caused by the following changes of parameters:
    - The upper bound of simple-connection weights is changed to 1 from 5.
    - The learning rate of the threshold, $\eta_{\theta}$, is changed to 1e-3 from 1e-2.
    - The number of epochs for training complex cells is changed to 4e6 from 1e6.
- Results
  - Used the natural video and generated updated figures for the entire Results section.
  - Modified corresponding text for updated figures.
  - Added a subsection ‘*Normalization gain determines the level of competition*’ to identify the key feature of the modified NBCM learning rule and transition from repetitious complex cells to diverse complex cells.
  - Combined different examples of complex cells into one figure for better illustration.
  - For the subsection ‘Population statistics compared with experimental data’, the comparison between experimental and model data was done using the Welch’s t-test and a percentage, *[overlap between histograms of model and experimental data] / [histogram spanned by both model and experimental],* to provide quantitative comparison between the model and experimental data.
- Discussion
  - Added a new section ‘*Natural video vs. jittered natural images*’.
  - Moved a few sentences into the subsection ‘*Normalization gain determines the level of competition*’ in the Results.
- Supporting information
  - S1 Appendix: put results generated using jittered images here.
  - S2 Appendix: update figures.
  - Removed some figures.
- References
  - Added a new reference for the computing platform on which the simulations were run.
  - Updated the detail of two references.
- Other small changes highlighted in the manuscript
  - Some typos and syntax errors are corrected, as identified by the reviewers.

Thank you for your consideration and we look forward to hearing from you.

Yours sincerely,

Yanbo Lian

On behalf of the authors: Yanbo Lian, Ali Almasi, David B. Grayden, Tatiana Kameneva, Anthony N. Burkitt, Hamish Meffin
