# Supplementary material for: Learning receptive field properties of complex cells in V1
Source: PLoS Comput Biol. 2021 Mar 2;17(3):e1007957. doi: 10.1371/journal.pcbi.1007957 (PMC7954310; doi:10.1371/journal.pcbi.1007957)
Supplement: Attachment — Submitted filename: Response Letter.docx [file pcbi.1007957.s005.docx]

Department of Biomedical Engineering

The University of Melbourne

Carlton, VIC 3053, Australia

[yanbo.lian@unimelb.edu.au](mailto:yanbo.lian@unimelb.edu.au)

18^th^ Dec. 2020

**Re: Response letter – PLOS Computational Biology (PCOMPBIOL-D-20-00804R1)**

Dear Deputy Editor and Reviewers,

Thank you for your comments on our revised manuscript, “Learning receptive field properties of complex cells in V1”. We have corrected some errors in syntax and made some changes to improve the presentation of the paper.

**In response to the comments of Reviewer #1**:

1) First, the paper is in general clearly written and would benefit for some simplification in the presentation of the results. First, I recommend to fully check the syntax. In particular, some sentences are more than three lines long and should be split. There a number of occurrences of missing articles (eg l550 " of response " ; l573 "Complex cell that" > "A complex cell that" ; l646 " introduces competition" ; ...). Also some terms could be better chosen, for instance l400 "how widely" -> "how broadly" (?); l487 "the *pronounced* simple cell"; l534 "moderate tuning ".

- Some long sentences have been split into shorter sentences. Corresponding changes in the manuscript:
  - Line 3-7: *‘They categorized simple cells as neurons that have receptive fields (RFs) with a spatial structure consisting of distinct light (ON) and dark (OFF) regions. Furthermore, the RFs of simple cells exhibit positive summation within ON and OFF regions and show antagonism between ON and OFF regions.’*
  - Line 12-14: *‘In contrast, complex cells exhibit significant nonlinear spatial integration. While they respond strongly to moving oriented edges, they do not show the other characteristics of simple cells described above.’*
  - Line 50-54: *‘From the perspective of a hierarchical structure, the energy model can be understood as a complex cell having convergent inputs from four simple cells with different spatial phase preferences. For example, the response of the complex cell is the weighted sum of four simple cells, as shown in Fig 1B.’*
  - Line 104-108: *‘Second, the weights connecting simple and complex cells are not learned in some models. The weights in the ISA model [28] are fixed, with only the weights of the simple cells learned. The weights in Hosoya and Hyvarinen's model [30] are computed by strong dimensionality reduction using Principal Component Analysis (PCA), which does not correspond to a form of synaptic plasticity.’*
  - Line 201-205: *‘The purpose of this step is to limit the spatial range of synaptic connectivity with LGN and, subsequently, V1 cells, similar to the approach used by Linsker [43]. It also puts more emphasis on the central part of the image patch to cause the learned simple RFs to be more centralized in the 2D image domain.’*
  - Line 461-465: *‘For example, orientation breadth is defined as the range of orientations of features from the cell's subspace to which the cell showed invariant response. This invariance for a given reference rate includes only features that required less than twice the contrast needed by the cell's most sensitive feature to attain that rate.’*
  - Line 534-536: *‘Furthermore, the scatter plots of F1/F0 vs. preferred orientation (right column of Fig 5B, C, and D) cover a much wider range compared with Fig 5A. They include all orientations (0 to 180) and …’*
- We have added missing articles. Corresponding changes in the manuscript:
  - Line 536: ‘range of’ -> *‘a range of’*
  - Line 559 and 673: ‘response’ -> *‘responses’*
  - Lines 583, 591 and 598: ‘Complex cell’ -> *‘A complex cell’*
  - Lines 722: ‘competition’ -> *‘the competition’*
- We have changed the use of some words in the paper. Corresponding changes in the manuscript:
  - Line 403: ‘how widely’ -> *‘how broadly’*
  - Lines 482, 484, 490, 501, 505, 507, 528 and 586, and Fig 5 caption: ‘pronounced’ -> *‘substantial’*
- We have added a short sentence in brackets after ‘moderate tuning’ to explain what we mean by ‘moderate’. Corresponding changes in the manuscript:
  - Line 536-538: *‘… and a range of spatial phase tuning from complete invariance (F1/F0 close to 0) to moderate tuning (F1/F0 between 0 and 1).’*

2) Second, the main contribution of the paper is to propose a novel model and to show how it models the learning of complex cells. instead of being "yet another model" it would be a great contribution to show the generality of their result and highlight the novelty in that model. In particular, having as a result that $\beta\approx12$ does not bring much to the community. Showing that having a smooth competition (through normalization) allows to have a better fit should be discussed. Another point is that saying that "efficient coding does not learn complex cells" is quite overstated. Efficient coding has many facets and in appendix S1, you study just that relative to your architecture.

- We have changed the result section, ‘Normalization gain determines the level of competition’, to *‘Normalization introduces smooth competition required to learn different complex cells’*. In this new result section, we have also added a paragraph discussing the normalization that introduces smooth competition. Along with ‘Response normalization’ and ‘Efficient coding’ in the “Discussion”, the presentation of the paper is clearer. Corresponding changes in the manuscript:
  - Line 540: section name ‘Normalization gain determines the level of competition’ -> *‘Normalization introduces smooth competition required to learn different complex cells’*
  - Line 542-548: *‘The competition between neurons is important for the network to learn diverse properties. Results presented above indicate that the competition introduced by the normalization in the NBCM rule promotes the diversity of tuning across the population of complex cells. However, based on the hierarchical assumption of the model, the competition introduced by efficient coding is too strong to produce RF properties of complex cells (see Discussion and S2 Appendix). This is also consistent with the result that the normalization gain, β, determines the level of competition.’*
  - Line 575-576: *‘Therefore, smooth competition that promotes diversity but preserves some invariance is crucial to the learned RF properties of complex cells.’*
- We have explicitly clarified in the paper that the investigation of efficient coding assumes a hierarchical structure and the manuscript has no sentence simply claiming “efficient coding does not learn complex cells” now. Corresponding changes in the manuscript:
  - Line 120-123: *‘Though efficient coding can learn simple cells, we found that a cascaded stage of efficient coding cannot effectively learn the RF properties of complex cells from simple cell responses (see Discussion and S2 Appendix for details).’*
  - Line 725-726: *‘Based on our assumption of a hierarchical structure, we conducted extensive investigation to understand if efficient coding, …’*

3) ‘Figs’ > ‘Fig’

- We have corrected this typo. Corresponding changes in the manuscript:
  - Lines 479 and 490: ‘Figs’ -> *‘Fig’*

Thank you for your consideration and we look forward to hearing from you.

Yours sincerely,

Yanbo Lian

On behalf of the authors: Yanbo Lian, Ali Almasi, David B. Grayden, Tatiana Kameneva, Anthony N. Burkitt, Hamish Meffin
